# The coexistence of wildlife and livestock

**DOI:** 10.1093/af/vfad064

**Published:** 2024-02-14

**Authors:** Patricia Barroso, Christian Gortázar

**Affiliations:** Department of Veterinary Sciences, Faculty of Veterinary, University of Turin, Grugliasco, Turin, Italy; SaBio Instituto de Investigación en Recursos Cinegéticos IREC (UCLM & CSIC), Ciudad Real, Spain

**Keywords:** Agroecosystem services, Conflicts, Habitat change, Herbivore dynamics, Wildlife-livestock interface

**Implications**

- Livestock impacts on the environment are positive or negative depending on the region, timeframe, stocking rate, and farming system. Wildlife can impact livestock farming through infection maintenance, predation, and competition. Thus, the coexistence of wildlife and livestock has multiple facets. This text examines conflicts and opportunities within this frame.
- Livestock grazing can cause woodland destruction or favor habitat diversity by generating and maintaining lentic waterbodies and limiting woody encroachment. In turn, woodland expansion can negatively affect livestock farming due to pasture loss and wild ungulate overabundance. Rangelands are valuable habitats that are vulnerable to fragmentation and land use change, with effects on their suitability for grazing-based livestock farming and on their contribution to biodiversity.
- Sources of wildlife-livestock conflict include shared infections, large predators and obligate scavengers, competition for food and water, and fencing. Mitigating these conflicts requires considering the interests of the relevant sectors, i.e., the human factor.
- Intervention options include zoning and land use planning, diversifying community livelihoods and lifting restrictions on wildlife harvest, establishing damage compensation and pasture fencing schemes, deploying biosafety measures to reduce wildlife-livestock contacts, and manipulating livestock densities and wild herbivore populations through farming and hunting for targeted use of chronic disturbance to improve ecosystem patterns and processes.
- We conclude that wildlife-livestock coexistence is a must, considering global concerns about food security, biodiversity, and diseases. Research in this complex, transdisciplinary field is urgently needed to find out how to maximize both food safety and ecosystem services while minimizing potential adverse effects.

## Background

Agricultural production ranks among the most environmentally impactful sectors at the global level, mainly through land use change and fertilizer use, and is estimated to contribute 10% to 11% of greenhouse gas (GHG) generation in the United States and the European Union. Globally, the consumption of animal-sourced food products is regarded one of the most powerful negative forces affecting the conservation of terrestrial ecosystems and biodiversity, especially because increasing livestock farming causes significant habitat loss in developing megadiverse tropical countries. Livestock production is a contributor to soil loss and nutrient pollution and has been linked to decreases of apex predators and wild herbivores (Tilman et al., 2001). Land use change is also regarded as a driver of pathogen emergence and the increase in heads of ruminants through time has been proposed to contribute to the number of infectious disease outbreaks registered and to the increase in number of threatened wildlife species (Morand, 2020), thereby linking animal farming, biodiversity conservation, and disease emergence.

However, semi-natural grazing areas provide food and support livelihoods for millions of people and contribute to social and ecological health and well-being (Godde et al., 2020). As opposed to tropical contexts, semi-natural grazing areas in the northern hemisphere are unfertilized pasturelands with a long history of traditional low-input grazing management. Regenerative grazing can increase plant biodiversity, build soils, sequester carbon, increase soil nitrogen and water content, and overall, increase productivity and sustainability (Teague et al., 2016). Since these biotopes are recognized for their high species richness, ruminant husbandry may have a satisfactory environmental performance in this region including positive impacts on biodiversity (Angerer et al., 2021) in line with the European Biodiversity Strategy for 2030 (https://environment.ec.europa.eu/strategy/biodiversity-strategy-2030_en). Therefore, natural grazing-based livestock farming systems can satisfy societal demands for public goods such as landscape and biodiversity.

**Figure 1.**
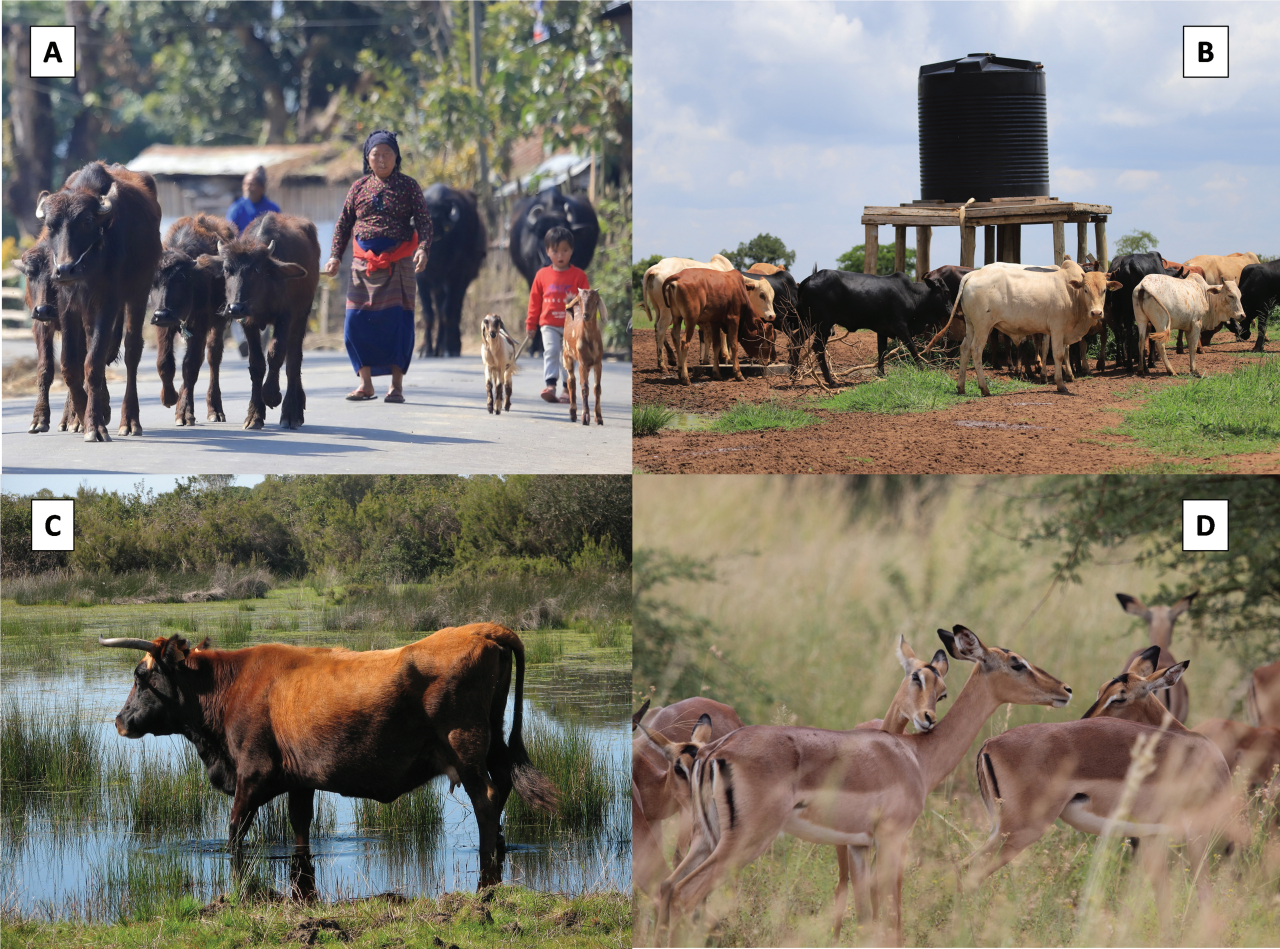
Multiple facets of wildlife-livestock coexistence. Livestock impacts on the environment can be positive or negative depending on the region and timeframe considered, and depending on stocking rates and farming systems. A: In tropical regions, primary forests and other undisturbed natural habitats are negatively impacted by their conversion into pastures or feed crops. Water buffalo and goats provide locals with meat, milk, hides and dung in the Nepal lowlands. B: Semi-natural grazing areas provide food and support livelihoods for millions of people and contribute to social and ecological health and well-being. Cattle belt, Uganda. C: Extensive cattle farming contributes to biodiversity conservation in regions with a long grazing history. Doñana National Park, Spain. D: Wildlife can negatively impact livestock interests through infection maintenance, predation, and competition for resources. Impalas, South Africa.

The paragraphs above evidence that livestock impacts on the environment are positive or negative depending on the geographical region (tropics vs. temperate regions) and timeframe considered. While primary forests and other undisturbed natural habitats are negatively impacted by their conversion into pastures or feed crops, livestock contributes to biodiversity conservation in regions with a long grazing history (de Santiago et al., 2022). Wildlife, in turn, can negatively impact livestock interests through infection maintenance, predation, and competition for resources. Thus, the coexistence of wildlife and livestock has multiple facets (Figure 1). This text examines conflicts and opportunities within this frame.

## Habitat Change and Livestock-Wild Herbivore Dynamics

**Figure 2.**
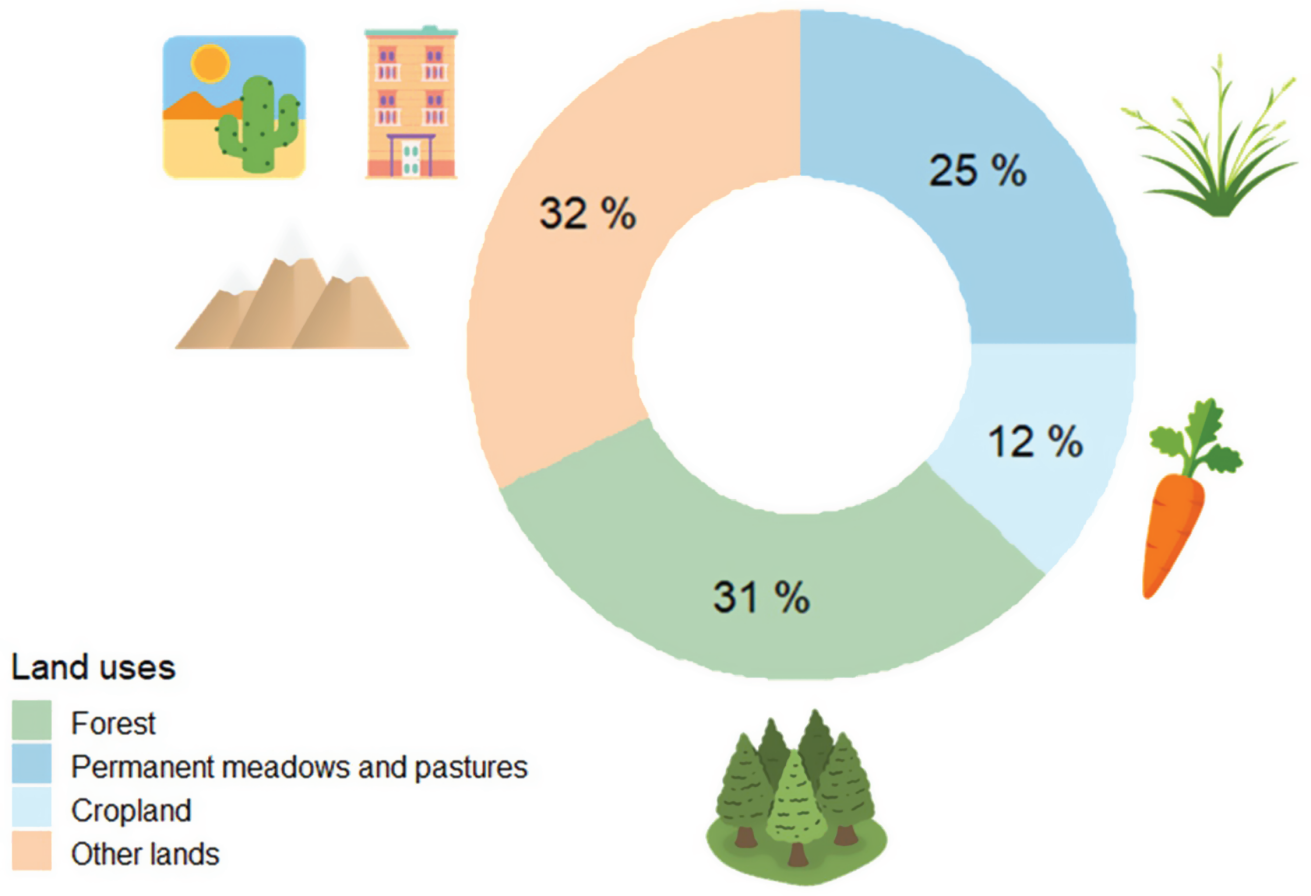
Proportion of the earth’s surface occupied by forest, agriculture lands (crops and pastures), and other lands, including urban habitats. Source: (FAO, 2021) (https://www.fao.org/faostat/en/#home). Livestock grazing occurs in several land types on more than one-third of the earth’s land surface (Fleischner, 1994).

Livestock grazing is a widespread land use which occurs in a broad diversity of ecosystem types in more than one-third of the earth’s land surface (Fleischner, 1994). Figure 2 presents the proportion of the earth’s surface occupied by forest, agriculture lands (crops and pastures), and other lands, including urban habitats. This distribution of land use is the consequence of 8,000 years of progressive change. These changes have effects on the dynamics of livestock and wildlife and vary depending on habitat type. Where wild ungulates and livestock compete for resources, mostly food, it is expected that population trends of both competing groups will correlate negatively (Vavra et al., 2004).

### Woodlands

The progressive loss of tropical rainforest is partly due to an increasing pressure to establish feed crops and livestock pastures and has dramatic consequences on biodiversity conservation (Morand, 2020). This is an ongoing process affecting megadiverse tropical regions worldwide. In temperate regions of Eurasia and North America, however, the decline of traditional agricultural practices including livestock grazing, among other drivers, may cause the opposite trend. This consists in woody encroachment or “shrubification” of pastures and other habitats, with negative effects on landscapes, farming, and biodiversity (Gellrich et al., 2007). Forests of the northern hemisphere are increasingly vulnerable to fire due to years of fire suppression, tree mortality, forest ingrowth, and less livestock grazing. Woodland and shrubland expansion, as well as declining numbers of grazing livestock, are drivers of wild ungulate overabundance. Both high stocking rates and wild herbivore overabundance are agents of chronic disturbance that have profound effects on ecosystem patterns and processes including nutrient cycles and succession (Vavra et al., 2004). In Europe and elsewhere, expanding wild ungulate populations also pose a threat to grazing-based livestock farming due to shared infections such as animal tuberculosis caused by the *Mycobacterium tuberculosis* complex (MTC; Gortázar et al., 2016). Thus, depending on the situation, livestock grazing can cause woodland destruction or favor habitat maintenance by limiting woody encroachment and creating and maintaining lentic waterbodies. In turn, woodland expansion can negatively affect livestock farming due to pasture loss and wild ungulate overabundance.

### Pasture ecosystems

Rangelands or dryland pasture ecosystems comprise between one-third and one-half of the land area in the world and maintain both pastoral economies and associated wildlife (Hobbs et al., 2008). Contrary to intensive agriculture activities, rangelands provide a suitable habitat for wildlife, even outside protected areas (FAO, 2009). Herbivory creates structural heterogeneity by removing herbaceous biomass and through browsing. The productivity of populations of wild and domestic herbivores depends on access to heterogeneity in landscapes, while fragmentation through processes such as barriers, decoupling, and contraction, reduces the ecosystem services of rangelands (Hobbs et al., 2008). Long-term research in the Serengeti semiarid savanna ecosystem in eastern Africa revealed that the Kenyan part of the Serengeti showed rapid land use change and drastic wildlife declines, while these changes were less pronounced on the Tanzanian side. This allowed to infer that changes in land cover and dominant grazer species numbers were driven primarily by private landowners responding to market opportunities for mechanized agriculture and less by agropastoral population growth or cattle numbers (Homewood et al., 2001). Rangelands are valuable habitats which are vulnerable to fragmentation and land use change, with effects on their suitability for grazing-based livestock farming and on their contribution to biodiversity.

## Livestock Effects on Biodiversity

**Figure 3.**
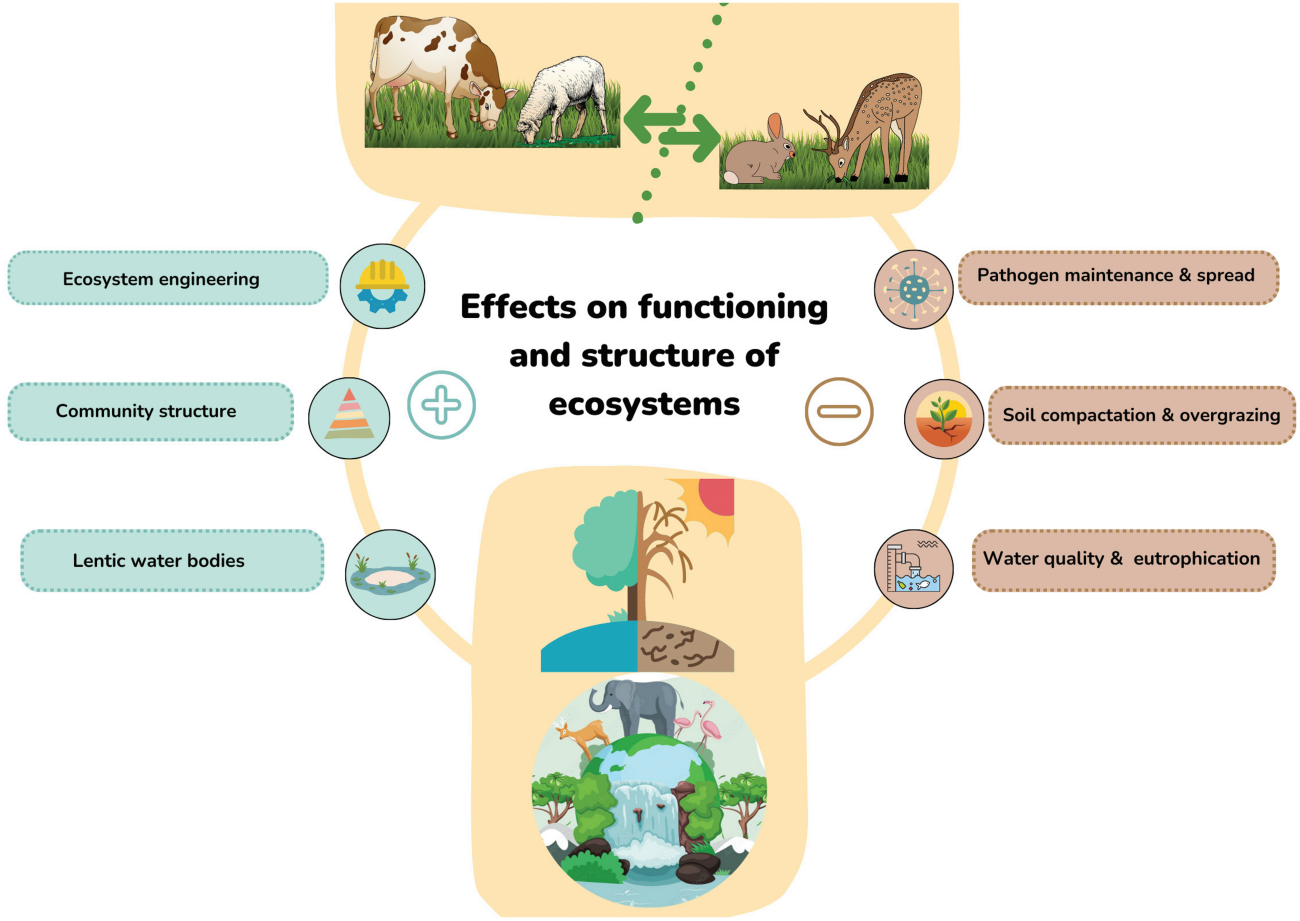
Potential effects of the coexistence of wildlife and livestock on the ecosystem and biodiversity.

The effect of grazing-based livestock farming on biodiversity can be both positive and negative (Figure 3; Gordon, 2018; Jori et al., 2021). For instance, high stocking rates or grazing intensity can overexploit the vegetation, cause soil loss, and alter the quality of waterbodies with subsequent effects on biodiversity (Broom et al., 2013). Furthermore, livestock can introduce pathogens and displace conflict wildlife into less favorable habitats (Jori et al., 2021). At the species community level, livestock grazing can cause alterations in species composition, decreased population density for certain taxa, or changes in community structure and organization. At the ecosystem level, livestock grazing can alter nutrient cycles and cause eutrophication, erosion, soil compaction and the subsequent decrease of water infiltration, or physical damage (trampling, rubbing, and browsing), with consequences for plant growth and habitat structure. These impacts could lead to cascading effects on forage quality and quantity, prey abundance, shelter availability and presence of suitable nesting or breeding areas, among others (Fleischner, 1994). The risks vary depending on farming systems and stocking rates and on the season and climate, the geographical region, and the indicator/target wild species considered (Broom et al., 2013; Schieltz and Rubenstein, 2016). Small mammals and ground-nesting birds are more susceptible, and diet and behavior are additional determinants of the expected impact derived from livestock. Thus, the costs or benefits of livestock grazing are species- and context-specific (Fulbright and Ortega-Santos, 2006).

On the positive side, it is well established that rangelands contribute significantly to global biodiversity. For instance, 50% of European bird species inhabit these areas (Pienkowski and Pain, 1997; Geiger et al., 2010). Livestock can act as ecosystem engineers and are used as a tool to improve rangelands for ecosystem health or habitat management (Schieltz and Rubenstein, 2016). At the species community level, livestock grazing can trigger targeted alterations of plant species composition by increasing light availability for germination and colonization of selected plant species, as well as create bare land sites for the regeneration of those species (Bakker et al., 2006). At the ecosystem level, livestock grazing may cause desirable changes in vegetation structure or control the biomass of both native and exotic plant species, preventing fires and leading to an increase of the nutritional quality of food and providing wildlife species with refuge from predators (Severson, 1990). Micromammals, birds, and reptiles benefit from certain changes in vegetation structure and cover while ungulates may be affected by induced changes in forage quantity and quality or competition. Small and lentic surface water bodies are valuable ecosystems with a critical contribution to biodiversity, resilience, and watershed functionality (Bolpagni et al., 2019). Consequently, the creation and maintenance of ponds on grazing-based farms benefits biodiversity (Thiere et al., 2009). In Mediterranean Spain, livestock farming maintains about 0.02 waterholes per square km and contributes significantly to bird and amphibian species richness. One challenge is to find the balance between the benefits of waterholes for biodiversity conservation and the risk they imply for persistence and transmission of pathogens that can pose a problem for livestock, but also for wildlife, such as MTC (Barasona et al., 2017).

Thus, although a negative effect of the presence of livestock on biodiversity might be expected, some authors reported that clever livestock management could reverse this trend (Dumont et al., 2013). The establishment of sustainable free-ranging livestock production with much greater biodiversity than in traditional systems and reduced land use could lead to healthier ecosystems and better animal welfare (Broom et al., 2013). This might imply adjusting the livestock species and the timing, intensity, frequency (rotational grazing), and selectivity of grazing (Vavra et al., 2004). In this regard, the concept “agricultural rewilding” may allow livestock systems to further mitigate their repercussion, restore biodiversity in ecosystems and provide more ecosystem services (Perino et al., 2019).

## Conflicts

**Figure 4.**
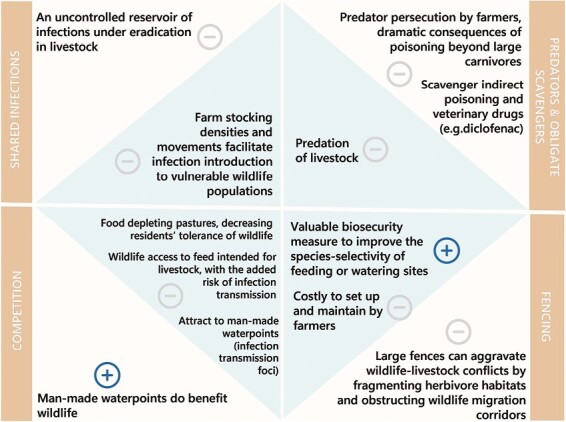
Positive and negative effects derived from the coexistence of livestock (internal triangles) and wildlife (external triangles) for each of the main identified conflicts.

Figure 4 displays the main conflicts derived from the coexistence of wildlife and livestock. These conflicts can be synthetized as follows:

### Shared infections

While pathogens are natural components of all ecosystems, infections shared between wildlife and livestock are prone to conflicts between stakeholders including farmers, hunters, and conservationists. It is noted that these stakeholder groups are not uniform and do include pro-farming conservationists as well as conservation-motivated farmers. These are often great conservationists and stewards of the land. Conflicts emerge where wildlife is perceived as an uncontrolled reservoir of infections under eradication in livestock (e.g., foot and mouth disease virus in African buffalo, African swine fever virus in wild boar, animal tuberculosis), or where farm animal densities and movements facilitate infection introduction to vulnerable wildlife populations (e.g., peste des petits ruminants virus and Mongolian saiga antelope). In consequence, modern disease control at the wildlife-livestock interface needs integrated approaches targeting complex domestic and wild host communities.

### Large predators, trophic rewilding, and obligate scavengers

Large predators rely on wild and domestic herbivores as staple prey, generating conflicts with livestock farmers both in subsistence farming and in gazing-based farming systems. Retaliatory predator persecution, eventually including the use of poison, has dramatic consequences reaching beyond large carnivores. In North America and Europe, apex predators and wild herbivores are increasing in range and number despite ongoing extensive ruminant farming (Chapron et al., 2014). However, conflicts are frequent, especially where predators are perceived as overprotected newcomers (wolf expansion, lynx reintroduction) and traditional mitigation tools such as shepherding, trained dogs or protected night enclosures have become less available.

Trophic rewilding is an ecological strategy that consists of species introduction in ecological assemblages to re-establish top-down trophic interactions and lost ecological processes such as trophic cascades (Svenning et al., 2016). The introduction of certain wild species is an increasingly used tool for conservation and climate change mitigation that can reduce biodiversity and ecological losses, but its consequences are controversial. For example, in the African savannah, the reintroduction of elephants inhibits woodland regeneration and promotes grasslands, which indirectly affects species composition and community dynamics (Gordon et al., 2023). The risks of rewilding include altered disease ecology with potential impact on human and livestock health as well as ecological and sociopolitical consequences.

Vultures are endangered obligate scavengers offering important ecosystem services. Vulture populations depend on open habitats and abundant carrion of domestic or wild mammals and are vulnerable to persecution, especially to poisoning which may target large carnivores, and to certain veterinary drugs such as the anti-inflammatory diclofenac. Solving this puzzle needs to consider farming and wildlife management with a broad perspective (Buechley and Şekercioğlu, 2016).

### Competition for food and water

Wildlife competes with livestock for food. Abundant wild ungulates can be perceived as depleting pastures and thus decrease residents’ tolerance of wildlife (Xu et al., 2020), and wildlife will also gain access to feed intended for livestock, with the added risk of infection transmission. While direct competition is less likely, water points attract both wildlife and livestock and have a two-sided effect (Gordon, 2018). On one side, man-made waterpoints do benefit wildlife and, on the other side, these can act as infection transmission foci.

### Fencing

Fencing is often used to prevent infection transmission or to protect pastures, crops, or farm premises. Small-scale fencing is a valuable biosecurity measure often used to improve the species-selectivity of feeding or watering sites. However, fences are costly to set up and maintain, and large fences can aggravate wildlife-livestock conflicts by fragmenting herbivore habitats and obstructing wildlife migration corridors (Gordon, 2018; Xu et al., 2020).

## The Human Factor at the Wildlife-Livestock Interface

The current increase of the earth’s surface dedicated to agriculture to cover the demands of human populations has led to an increased level of interaction between humans, livestock and wildlife (König et al., 2020; Pozo et al., 2021). Social conflicts derived from these interactions are extended around agricultural landscapes, protected areas, and human-associated landscapes (urban and peri-urban areas), where the attitudes toward human-wildlife overlap vary significantly depending on cultural perception and individual attitudes, socioeconomic factors, risk estimation and the wild species implicated (Jordan et al., 2020). These conflicts arise from the confrontation between the interests of agriculture and wildlife conservation, being often transformed into debates between different stakeholder groups with contrary interests (König et al., 2020). Despite the efforts to fulfil the interests of both sectors, decision-making could become challenging since the sociopolitical setting is essential for the effective management of these conflicts (Treves et al., 2009). The human-wildlife interactions could carry positive or negative consequences for the actors implicated since this is a reciprocal process (Soulsbury and White, 2015). However, these costs and benefits are unevenly distributed between rural/developing and urban/developed communities (“co-existence inequalities”; Jordan et al., 2020), the costs being higher for rural areas and developing countries.

Benefits are harder to quantify comprising several ecosystem services provided by wildlife. These wild species may favor biodiversity because of their role as ecosystem engineers. In addition, the human-wildlife interaction could also benefit human health and well-being (e.g., pleasure to encounter a wild animal), and contribute to pest species control and the subsequent potential improvement of agricultural outputs (Soulsbury and White, 2015).

## Intervention Opportunities

Interventions for wildlife-livestock conflict mitigation include (1) zoning and land use planning, (2) diversifying community livelihoods, (3) damage compensation schemes and pasture fencing, (4) biosafety measures to reduce wildlife-livestock contacts, and (5) manipulating livestock densities and wild herbivore populations through farming and hunting for a targeted use of chronic disturbance to improve ecosystem patterns and processes. Further, (6) lifting restrictions on the use of (state-owned) wildlife might contribute to avoiding the progressive replacement of wildlife by (private-owned) livestock (Homewood et al., 2001; Vavra et al., 2004; Xu et al., 2020).

In this regard, it would be important to harmonize local concerns on food security and economy with international interests for conservation, enabling the effective management of conflicts without affecting biodiversity (Treves et al., 2006). To this purpose, it is necessary to address inequality (improve the distribution of the costs across communities) and intolerance (drivers of communities’ perceptions of wildlife conflicts), considering the different social, economic, and ecological/environmental contexts (Jordan et al., 2020). Finally, bridging the gap between science and practice by combining technical expertise with local knowledge would help to reach a co-existence status. The main tool available to achieve these goals is the application of interdisciplinary socioecological dynamic approaches (König et al., 2020; Pozo et al., 2021). Co-management, i.e., the cooperation of affected groups, government agencies and, potentially, a third impartial party, could be key in dealing with human-wildlife conflicts (Treves et al., 2006).

## Conclusion

The sustainability of natural resource-based livestock farming in relation to global concerns about climate change, biodiversity, diseases, and the quality of the agro-ecosystem services that are provided to society and their trade-offs has become a fundamental issue for public and scientific debate. Research in this complex, transdisciplinary field is urgently needed to find out how to maximize food security and ecosystem services while minimizing adverse effects.

## About the Authors

**Dr. P. Barroso** is a researcher for the Department of Veterinary Sciences of the University of Turin. Main research activities include health and population monitoring of wildlife, eco-epidemiology of shared pathogens at the wildlife-livestock-human interface, and disease control. Main research topics include the long-term dynamics and epidemiology of shared pathogens in complex host communities, the design of integrated wildlife monitoring systems and the study of biodiversity–disease relationships.

**Professor C. Gortázar** heads the Health and Biotechnology (SaBio) group at the National Wildlife Research Institute IREC (Universidad de Castilla La Mancha & CSIC) in Ciudad Real, Spain. His main research activities combine ecology, veterinary sciences, and biotechnology to address global challenges in health and conservation.
